# Precision Denavit–Hartenberg Parameter Calibration for Industrial Robots Using a Laser Tracker System and Intelligent Optimization Approaches

**DOI:** 10.3390/s23125368

**Published:** 2023-06-06

**Authors:** Mojtaba A. Khanesar, Minrui Yan, Mohammed Isa, Samanta Piano, David T. Branson

**Affiliations:** Faculty of Engineering, University of Nottingham, Nottingham NG7 2RD, UK

**Keywords:** positional accuracy, industrial robots, collaborative robots, Denavit–Hartenberg parameter calibration, forward kinematic calibration, laser tracker system, artificial intelligence for optimization

## Abstract

Precision object handling and manipulation require the accurate positioning of industrial robots. A common practice for performing end effector positioning is to read joint angles and use industrial robot forward kinematics (FKs). However, industrial robot FKs rely on the robot Denavit–Hartenberg (DH) parameter values, which include uncertainties. Sources of uncertainty associated with industrial robot FKs include mechanical wear, manufacturing and assembly tolerances, and robot calibration errors. It is therefore necessary to increase the accuracy of DH parameter values to reduce the impact of uncertainties on industrial robot FKs. In this paper, we use differential evolution, particle swarm optimization, an artificial bee colony, and a gravitational search algorithm to calibrate industrial robot DH parameters. A laser tracker system, Leica AT960-MR, is utilized to register accurate positional measurements. The nominal accuracy of this non-contact metrology equipment is less than 3 μm/m. Metaheuristic optimization approaches such as differential evolution, particle swarm optimization, an artificial bee colony and a gravitational search algorithm are used as optimization methods to perform the calibration using laser tracker position data. It is observed that, using the proposed approach with an artificial bee colony optimization algorithm, the accuracy of industrial robot FKs in terms of mean absolute errors of static and near-static motion over all three dimensions for the test data decreases from its measured value of 75.4 μm to 60.1 μm (a 20.3% improvement).

## 1. Introduction

Industrial robots are vital pieces of equipment for completing a modern manufacturing process. An industrial robot integrates several advanced technologies including mechanics, electronics, interfaces, and control in its structure [1]. To perform precision positioning [2], assembly, peg-in-hole [3,4,5,6,7], object handling, and manipulation on a factory floor [8], it is required to integrate accurate robot forward kinematics (FK) into its control methodology [9,10,11]. There exist some uncertainties in industrial robot FKs, which decrease its operational accuracy. Zero offset [12], irregularities in industrial robot geometry, robot manufacturing tolerances, assembly tolerances, structural deformations, and environmental factors are major sources of uncertainty in industrial robot FKs. Therefore, to increase the precision of an industrial robot FK, it is required to perform a calibration procedure [13,14]. The FK calibration is the procedure of improving the precision of the industrial robot Denavit–Hartenberg (DH) parameters to improve the robot FK model to have better positional accuracies [15,16]. The calibration of industrial robots is performed in multiple levels.

Three different calibration levels have already been identified for industrial robots [17]. The first calibration level (level I) is the one associated with robot joint angle encoders. The purpose of calibration in this level is to find a correcting relationship between the industrial robot joint angle readings from the installed joint angle transducer and the actual joint angle values. The second level (level II) of calibration is the one associated with the FK model, which is mainly due to parameter uncertainties and industrial robot geometry. The purpose of the calibration at this level is to estimate the true FK parameters as well as to determine the basic kinematic geometry [17]. Non-kinematic calibration is the calibration at level III, which is mostly carried out to compensate for robot structural flexibilities as well as joint compliance, link compliance, friction, and clearance. The calibration is performed at level II in this paper; it is required to process real-time data from industrial robots and form independent measurement equipment to improve positioning precision. The heterogeneous information gathered from multiple measurement systems increases the perception capability of the overall calibration system. Industrial robot FK calibration can be performed using parametric robot calibration and non-parametric robot calibration.

The majority of industrial robot FK errors are due to structural parameter measurement errors [18,19]. There are two types of industrial robot calibration categories: parametric robot calibration and non-parametric robot calibration. Non-parametric robot calibration techniques include bilinear and fuzzy interpolation methods as well as neural network approaches. The 3D position calibration using non-parametric approaches with different non-contact metrology equipment such as the Leica SMART310 laser tracker, Leica AT960, and Leica AT960-MR has already been performed for a PA10 robot arm, IRB1410, and a collaborative industrial robot, respectively [20,21,22]. A similar approach is used in [15,20] for the calibration purpose of a Hyundai HH800 robot, a heavy duty industrial robot, using a laser tracker system. Deep neural networks have been used to calibrate a KUKA KR500, an SRA166 using an API Radian laser tracker, and a laser tracker manufactured by IHI Scube, respectively [23,24]. Motivated by the fact that parametric calibration algorithms do not necessitate the use of a highly complex system such as a neural network to obtain the 3D position of robots, in this paper, parametric calibration algorithms are used to have an estimation of the real physical parameters of industrial robots. Furthermore, using the parametric approaches, it is possible to come up with a more compact and less complex type of mathematical equation. To perform the parametric calibration, it is formulated as an optimization algorithm which is solved using four different optimization algorithms from the three main optimization categories of evolutionary optimization algorithms, namely, swarm intelligence and physic-inspired optimization algorithms.

Evolutionary optimization, swarm intelligence, and physic-inspired optimization algorithms are the three major intelligent optimization algorithms preferred for the case when the cost function is not defined in an explicit form [25]. In the case when the function to be optimized is non-differentiable and/or not explicitly defined, intelligent optimization approaches may be more preferable than classical optimization methods. Moreover, meta-heuristic approaches benefit from multiple start points and information exchange between the solutions, which makes them overcome the local minima problem. While evolutionary optimization algorithms rely on Darwin’s natural selection principles [26], swarm intelligent [27,28] techniques imitate the chaotic and unpredictable motion behavior of species such as birds and fish in a swarm to perform optimization. An artificial bee colony (ABC) is another strong optimization approach which imitates the behavior of bees within their colony, and hence, they belong to swarm intelligence approaches. On the other hand, physic-inspired optimization algorithms use models of physical laws such as gravitational forces [29], coulomb forces [30], and quantum physics principles [31] to perform optimization. Inspired by the well-established results obtained from differential evolution [32] (DE), particle swarm optimization (PSO) [27,28], ABC [33,34], and gravitational search algorithms (GSA) [13], these four algorithms are used in this paper to perform industrial robot DH parameter optimization. Although the three main categories of optimization differ substantially in their metaphor, in this paper, they are used for the optimization purpose of a single cost function. A comparison is made between the results obtained from each of these optimization algorithms. Using a diverse range of intelligent optimization approaches makes the comparison results more reliable.

To perform the calibration of industrial robots, four optimization algorithms of DE, PSO, ABC, and GSA are utilized. The experimental setup in this paper consists of a laser tracker and collaborative robot. The laser tracker used in this study is a Leica AT960, a contactless metrology device capable of performing the 3D absolute position measurement of its retroreflector using laser interferometry principles. The positional precision of Leica AT960 is 3 μm/m (https://www.hexagonmi.com/-/media/Hexagon%20MI%20Legacy/m1/metrology/general/brochures/Leica%20AT960%20brochure_en.ashx (accessed on 1 May 2022)), and its maximum measurement volume is up to 40 m. The absolute position measurements from the laser tracker are used to estimate the robot DH parameters. The joint angle readings from the industrial robot are used within its FK to calculate the industrial robot position. However, the positions calculated from the joint angles of the robot contain uncertainty because of the uncertainty in the industrial robot DH parameters. A cost function is defined based on the error between the FK robot positions and the measured position from a laser tracker. The data points selected for this experiment correspond to the static and near static motion of the industrial robot. Data are split to train and test to validate the accuracy of the results. The cost function is then optimized using the four optimization algorithms of DE, PSO, ABC, and GSA. DE belongs to the evolutionary optimization category of optimization, PSO and ABC belong to the swarm intelligence optimization category, and GSA is a physics-inspired optimization algorithm. The results of these optimizations give the precise DH parameters of the industrial robot. The comparison made between the results obtained using these algorithms reveals that ABC outperforms DE, PSO, and GSA in terms of the mean square value of the calibration error. It is further observed that, by using ABC, it is possible to decrease the mean absolute error of industrial robot positions for the test data from 75.4 to 60.1 μm, which is a 20.3% improvement in accuracy. The results reveal that ABC results in a superior performance compared to DE, PSO, and GSA for industrial robot DH parameter estimation purposes.

This paper is organized as follows: in Section 2, the overall methodology including an industrial robot FK, intelligent optimization methods, and the proposed calibration approach are introduced. The experiment setup used for measurements is presented in Section 3. Experimental results are presented in Section 4. Section 5 concludes the paper.

## 2. Methodology

**Figure 1:**
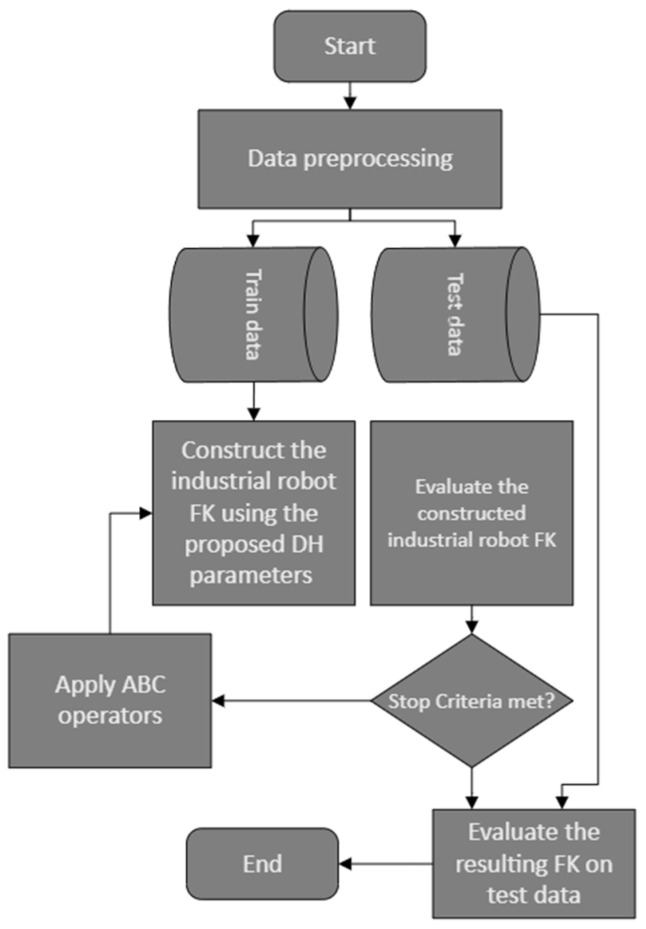
Overall calibration flowchart.

The overall calibration process, which implements metaheuristic optimization approaches to perform calibration, is presented in this section (see Figure 1). Each element in this flowchart is presented in detail in the next few sections. Although joint angle readings from an industrial robot can be used to find its end effector position, uncertainties associated with industrial robot DH parameters, obtained from its geometry, result in positional inaccuracies. The manufacturing tolerance leads to differences in DH parameter values from one robot to another of a similar build. In this paper, a laser tracker system, Leica AT960-MR, is utilized to increase the accuracy of DH parameter values.

### 2.1. Data Preprocessing

This laser tracker is capable of measuring accurate non-contact robotic positions at a maximum distance of 60 m. In this work, the laser tracker is used within 2.8 m from the robot base. It uses laser interferometry principles and internal precise encoders for positioning. The 3D position readings from the robot are used for DH parameter calibrations using metaheuristic optimization approaches. The position reading from the industrial robot is conducted at a sampling frequency of 125 Hz, and the sampling frequency of the laser tracker system is 10 Hz. To perform calibration, it is required to synchronize the measurements from the industrial robot joint angle encoders and resample them at the same time instances as those for the laser tracker system. It is further required to extract samples occurring at a linear velocity of less than 2 mm/s using the nominal DH parameters of UR5 to have a quasi-static calibration for the robot. Overall, we have 209 data points which are split into train and test data using a 70/30 ratio. The resulting synchronized data values are then used with a metaheuristic optimization approach to calibrate the DH parameters of industrial robots. The details of the overall calibration approach are presented in this section.

### 2.2. FK Model of UR5

**Figure 2:**
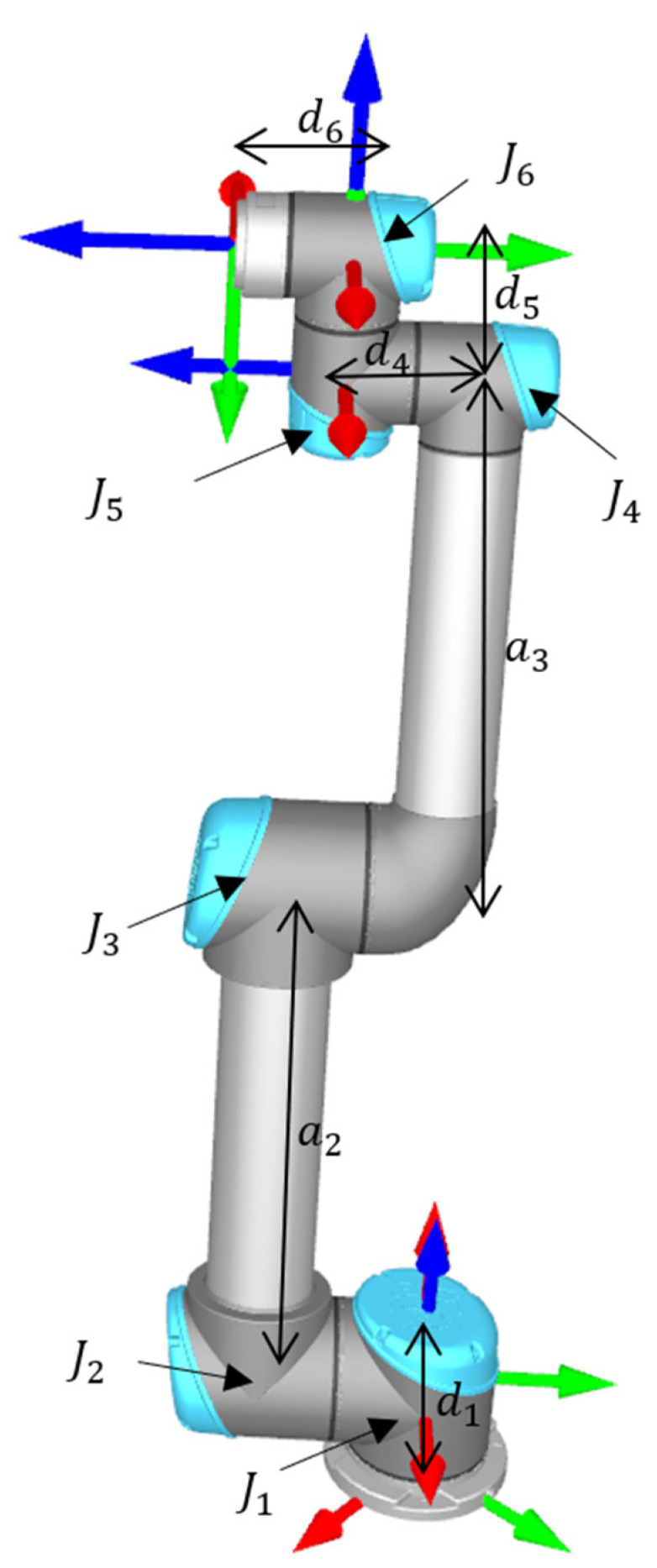
Typical industrial robot (UR5) schematics $(d_{i},i=1,4,5,6$ and $a_{i},i=2,3$ parameters refer to the DH parameters of the UR5).

The mathematical function operating on industrial robot joint angles to find the Cartesian coordinates of an industrial robot within a 3D workspace is called FKs. The link transformation matrix from the link i−1 to the link i using the DH parameters of the robot, as in Table 1, depends on the corresponding joint angle of the industrial robot and its other DH parameters [35,36] (see Figure 2).

(1) Tii−1=cqi−cαisqisαisqiaicqisqicαicqi−sαicqiaisqi0sαicαidi0001

where $q_{i}\prime s,i=1,\ldots ,6$ represents the joint angle i, $\alpha_{i}\prime s,i=1,\ldots ,6$, $a_{i}\prime s,i=1,\ldots ,6$ and $d_{i},i=1,\ldots ,6$ represents the other DH parameters of the robot. Furthermore, ${cq}_{i},{sq}_{i},c\alpha_{i},\;\mathrm{and}\;s\alpha_{i},i=1,\ldots 6$ represent ${cos(q}_{i}),$ ${sin(q}_{i}),$ $c{os\alpha }_{i},$ and $sin(\alpha_{i}),i=1,\ldots ,6$, respectively. Other than the DH parameters of the industrial robot in Figure 1, the labels $J_{i},i=1,\ldots ,6$ represent the six joints of the industrial robot. Overall, the robot transformation matrix in the robot base coordinates is obtained as follows [37].

(2) Te=T60=T10T21T32T43T54T65

The end effector positions in all three dimensions in the industrial robot coordinate are obtained as follows.

(3) $$x_{rr}=d_{4}s_{1}+a_{2}c_{1}c_{2}+d_{6}c_{5}s_{1}+a_{3}c_{1}c_{2}c_{3}-a_{3}c_{1}s_{2}s_{3}+d_{5}c_{1}c_{2}c_{3}s_{4}\;+d_{5}c_{1}c_{2}s_{3}c_{4}+d_{5}c_{1}s_{2}c_{3}c_{4}-d_{5}c_{1}s_{2}s_{3}s_{4}-d_{6}c_{1}c_{2}c_{3}c_{4}s_{5}+d_{6}c_{1}c_{2}s_{3}s_{4}s_{5}\;+d_{6}c_{1}s_{2}c_{3}s_{4}s_{5}+d_{6}c_{1}s_{2}s_{3}c_{4}s_{5}$$

(4) $$y_{rr}=a_{2}s_{1}c_{2}-d_{6}c_{1}c_{5}-d_{4}c_{1}+a_{3}s_{1}c_{2}c_{3}-a_{3}s_{1}s_{2}s_{3}+d_{5}s_{1}c_{2}c_{3}s_{4}\;+d_{5}s_{1}c_{2}s_{3}c_{4}+d_{5}s_{1}s_{2}c_{3}c_{4}-d_{5}s_{1}s_{2}s_{3}s_{4}-d_{6}s_{1}c_{2}c_{3}c_{4}s_{5}+d_{6}s_{1}c_{2}s_{3}s_{4}s_{5}\;+d_{6}s_{1}s_{2}c_{3}s_{4}s_{5}+d_{6}s_{1}s_{2}s_{3}c_{4}s_{5}$$

(5) $$z_{rr}=d_{1}+a_{2}s_{2}+a_{3}c_{2}s_{3}+a_{3}s_{2}c_{3}-d_{5}c_{2}c_{3}c_{4}-d_{5}c_{2}s_{3}s_{4}{+d}_{5}c_{2}s_{3}s_{4}\;+d_{5}s_{2}c_{3}s_{4}+d_{5}s_{2}s_{3}c_{4}-d_{6}c_{2}c_{3}s_{4}s_{5}-d_{6}c_{2}s_{3}c_{4}s_{5}-d_{6}s_{2}c_{3}c_{4}s_{5}\;+d_{6}s_{2}s_{3}s_{4}s_{5}$$

Although the values of the FK parameters are unknown and will be estimated, their numerical values, according to the robot manufacturer, are as follows (https://www.universal-robots.com/articles/ur/application-installation/dh-parameters-for-calculations-of-kinematics-and-dynamics/ (accessed on 1 May 2022)).

(6) $$d_{2}=d_{3}=0,\;\mathrm{and}\;a_{1}=a_{4}=a_{5}=a_{6}=0$$

(7) $$d_{1}=0.08916m,a_{2}=-0.425m,a_{3}=-0.392m,\;d_{4}=0.1092m,d_{5}=0.0947m,d_{6}=0.0823+d$$

where d is the distance between the center of the retroreflector and the center of the robot end-effector, which is measured as equal to 0.1695m. Furthermore, to conduct the calibration, the end-effector of the robot is considered facing down, with its TCP axis-rotation vector being equal to $\left(\begin{array}{ccc} \pi & 0 & 0 \end{array}\right)$.

### 2.3. Formulating the Estimation Problem as a Cost Function

It is required to define the industrial robot DH parameter estimation problem as a cost function to be optimized. This is a function of the industrial robot DH parameter and returns a quadratic function of the industrial robot FK error. Using each set of DH parameters given by each of the three optimization algorithms, DE, PSO, and GSA, the industrial robot FK is constructed. The positions given by the industrial robot FK are within its coordinate, which is the fixed coordinate attached to its base. The change in the coordinate is required to have the industrial robot positions in the laser tracker coordinates.

(8) $$\left[\begin{array}{c} x_{rl}\\ y_{rl}\\ z_{rl} \end{array}\right]={Tr}_{rl}\left[\begin{array}{c} \begin{array}{c} x_{rr}\\ y_{rr} \end{array}\\ \begin{array}{c} z_{rr}\\ 1 \end{array} \end{array}\right]$$

where $x_{rl}$, $y_{rl}$, and $z_{rl}$ are the robot end-effector positions using a laser tracker in a laser tracker coordinate, and ${Tr}_{rl}$ is the transformation matrix from the robot base coordinate system to the coordinate system of the laser tracker. The transformation matrix ${Tr}_{rl}$ can be easily calculated using a least squares algorithm [38]. Using the calculated transformation matrix ${Tr}_{rl}$, the end effector positions in laser tracker coordinates are calculated as follows.

(9) $$\left[\begin{array}{c} {x\prime }_{rl}\\ {y\prime }_{rl}\\ {z\prime }_{rl} \end{array}\right]={Tr}_{rl}\left[\begin{array}{c} \begin{array}{c} x_{rr}\\ y_{rr} \end{array}\\ \begin{array}{c} z_{rr}\\ 1 \end{array} \end{array}\right]$$

where ${x\prime }_{rl}$, ${y\prime }_{rl}$, and ${z\prime }_{rl}$ represent the robot end effector position in the *x*, *y*, and *z* axes using robot joint encoders in laser tracker coordinates. The position errors (er) can be calculated for each point as the difference between the robot end effector position using joint encoder data in the laser coordinate and the robot end effector measurements from the laser tracker.

(10) $$er=\sqrt{{\left({x\prime }_{rl}-x_{rl}\right)}^{2}+{\left({y\prime }_{rl}-y_{rl}\right)}^{2}+{\left({z\prime }_{rl}-z_{rl}\right)}^{2}}$$

The overall mean value of position errors for all measured points associated with each of these industrial robot FKs needs to be calculated using (11). The mean absolute position errors over all measured points are used to construct the cost function for each object in the intelligent optimization algorithm.

(11) $$C=\frac{1}{N_{m}}\sum_{i=1}^{N_{m}}{er}_{i}$$

where $N_{m}$ is the total number of measured points. The metaheuristic optimization procedure is then used to find the optimal values of the industrial robot DH parameters.

### 2.4. Metaheuristic Optimization Algorithms

Metaheuristic approaches are generally designed to solve complex optimization problems where the cost function is not explicitly given and/or suffers from multiple local minima. In this paper, four metaheuristic optimization algorithms of DE, PSO, GSA, and ABC are briefly explained, and they are used to estimate industrial robots’ DH parameters.

#### 2.4.1. Differential Evolution

Evolutionary algorithms are frequently used as metaheuristic optimization approaches to solve complex optimization problems. These algorithms are inspired by the natural selection principles and the Darwinian theory of evolution. Individual members of the evolutionary algorithm represent solutions for the optimization problem. In each generation of these algorithms, operators such as crossover, mutation, and selection are applied to the individuals to generate the next generation. The selection operator selects the individuals for crossover or mutations using their fitness function and some random operators. Crossover is an operator that is applied to two or more selected individuals to generate new generations as their mathematical combination. Mutation, on the other hand, is an operator that acts as a single individual to generate a new individual. The mutation operator avoids premature convergence by mutating individuals.

Candidate solutions are presented by a six-dimensional vector $X_{DE}^{i}=\left(d_{1}^{i},a_{2}^{i},a_{3}^{i},d_{4}^{i},d_{5}^{i},d_{6}^{i}\right),i=1,\ldots ,N$. Individuals representing solutions to the optimization problem need to be uniformly distributed between the permeable minimum and permeable maximum value of the solutions within each dimension. The mutation operator acts on the best individual by adding a term using two other randomly selected individuals from the population as follows [32]:

(12) $${V_{DE}^{i}\left(t\right)=X}_{DE,best}^{}\left(t\right)+F\left(X_{DE}^{r_{1}^{j}}\left(t\right)-X_{DE}^{r_{2}^{i}}\left(t\right)\right)$$

where $V_{DE}^{i}$ is the mutant vector, $X_{DE,best}^{}$ is the best individual member of DE, and the parameter F is the scale factor, which is selected as equal to 0.5 in this paper. Furthermore, *t* refers to the number of generations. The indices $r_{1}^{j}$ and $r_{2}^{i}$ are mutually exclusive integers randomly generated within the range [1,N], where N is the total number of individuals.

After performing the mutation operation, the crossover operation is applied to each individual and its mutant one [32].

(13) $$U_{DE}^{i,j}(t)=\left\{\begin{array}{cc} V_{DE}^{i,j}(t) & if\;{rand}_{j}[0,1]\leq CR\\ X_{DE}^{i,j}(t) & otherwise \end{array}\right.,j=1,\ldots ,d$$

where $X_{DE}^{i,j}(t),U_{DE}^{i,j}(t),$ and $V_{DE}^{i,j}(t)$ are the *j*-th element of $X_{DE}^{i}(t),U_{DE}^{i}(t),$ and $V_{DE}^{i}(t)$, respectively. $U_{DE}^{i}$ is the result of the crossover. The selection operator performs a comparison between the cost function associated with $X_{DE}^{i}$ and $U_{DE}^{i}$, and the result of the selection procedure is used to perform the crossover and mutation to generate the next generation [32].

(14) $$X_{DE}^{i}\left(t+1\right)=\left\{\begin{array}{cc} U_{DE}^{i}\left(t\right) & if\;f\left(U_{DE}^{i}\left(t\right)\right)\leq f\left(X_{DE}^{i}\left(t\right)\right)\\ X_{DE}^{i,j} & otherwise \end{array}\right.,j=1,\ldots ,d$$

The mutation, crossover, and selection steps are repeated iteratively until the optimization termination condition of the maximum number of iterations is met. The maximum number of iterations considered in this paper is equal to 300.

#### 2.4.2. Particle Swarm Optimization

The particle swarm optimization algorithm is based on swarm intelligence. This algorithm is developed by observing the behavior of fish and birds in a swarm [28]. The solutions to the optimization problem are presented as a position vector in a swarm. The velocity vector determines the change in the swarm position vector for the next iteration. The updates in the velocity vector are performed using the best personal experience of each swarm member and the best experience over the whole swarm. A term preserving the inertia of movement exists in the velocity vector update equation to add more exploration features to the swarm. To apply PSO to calibrate industrial robot FKs, the position vector in PSO is associated with the DH parameters as follows [28]:

(15) $$X_{PSO}^{i}=\left(d_{1}^{i},a_{2}^{i},a_{3}^{i},d_{4}^{i},d_{5}^{i},d_{6}^{i}\right)$$

where $X^{i}$ refers to the solutions within PSO and *i* refers to the *i*-th particle within the swarm.

The positions in the next generation of PSO using its current position vector and velocity vector are updated as follows [28]:

(16) $$V_{PSO}^{i}\left(t+1\right)=wV_{PSO}^{i}\left(t\right)+r_{1}c_{1}\left({pbest}_{i}\left(t\right)-X_{PSO}^{i}\left(t\right)\right)+r_{2}c_{2}\left({gbest}_{i}\left(t\right)-X_{PSO}^{i}\left(t\right)\right),i=1,\ldots ,N$$

(17) $$X_{PSO}^{i}\left(t+1\right)=X_{PSO}^{i}\left(t\right)+V_{PSO}^{i}\left(t\right)$$

where *t* refers to the current iteration, ${pbest}_{i}\left(t\right)$ presents the personal best experience of the *i*-th particle, ${gbest}_{i}(t)$ represents the overall best experience within the swarm, ${0<c}_{1},c_{2}$ are the two positive constants, and $r_{1},r_{2}$ are two uniform and random numbers from the interval of [0,1]. The parameter $c_{1}$ is the coefficient associated with the best personal experiment of the particles in the swarm, and the parameter $c_{2}$ is the coefficient associated with the best global experiment of the particles within the swarm. The parameter w is the inertia weight, which makes the swarm follow its previous search direction. The stability criteria for PSO require the following condition to be valid for its parameters [39,40].

(18) $$c_{1}+c_{2}<\frac{4\left(1-2w+w^{2}\right)}{1+w},\;0<w$$

It is further observed in [41] that while a large value for w improves exploration, a small value guarantees a good exploitation capability for PSO.

#### 2.4.3. Artificial Bee Colony

The ABC algorithm is designed to imitate the principle of foraging, which explains how bees work together to search to find the best hive and maximize the colony yield [33]. In an ABC algorithm, employed bees, onlookers, and scout bees are the three groups of bees which exchange information to perform optimization [34]. The main step of this algorithm is summarized as follows.

1. initialize the population as $x_{i},i=1,\ldots ,SN$
2. calculate the fitness associated with each member of the population
3. repeat the following loop:
  a. produce a new set of industrial robot DH parameters as the solutions for the optimization problem using the employed bee using $v_{ij}=x_{ij}+\varphi_{ij}\left(x_{ij}-x_{kj}\right),k\;\epsilon \;1,\ldots ,SN,j\;\epsilon \;1,\ldots ,6,$ where $\varphi_{ij}\;\epsilon \;[0,1]$ is a uniform random number
  b. calculate the fitness function associated with each solution ${f(x}_{i})$
  c. for each solution, calculate its selection probability value as follows:
    $$p_{i}=0.04\frac{{f(x}_{i})}{{max}_{i=1}^{SN}{f(x}_{i})}+0.96$$
  d. produce the new solutions $v_{i}$ for the onlookers from the solutions $x_{i}$ selected depending on $p_{i}$ and evaluate them
  e. apply a greedy selection process for onlookers
  f. find possible abandoned food sources for scouts and replace them with a new food source using $x_{ij}=x_{i,min}+r_{j}\left(x_{i,max}-x_{i,min}\right)$, where $r_{j}\;\epsilon \;[0,1]$ is a uniform and random number
  g. compare the best solution in this iteration with the overall best solution and replace it, if necessary
  h. if the maximum number of iterations ($T_{max}$) is achieved, stop; otherwise, continue the loop

#### 2.4.4. Gravitational Search Algorithm

Other than the swarm intelligence and evolutionary optimization algorithms, there exists a third class of intelligent optimization approaches inspired by physics. GSA is an algorithm in this category inspired by the Newtonian gravitational forces between objects. In this algorithm, each solution is defined as a position of an object. The object positions are updated using velocity and acceleration vectors. Each object in this optimization algorithm benefits from a mass property. The larger mass value is assigned to the solution with a better cost function. The acceleration vector at each iteration is updated such that the objects with smaller weights are accelerated towards the ones with larger weights. This makes the objects with a smaller mass value scan the solution space towards the objects with a larger mass value. After a few iterations, all solutions converge to the object with a larger mass value, which will conclude the optimization. The mathematical description of the algorithm is explained within the next paragraphs.

Each object in this algorithm benefits from several properties of mass, positions, velocity, and accelerations. Solutions in a six-dimensional solution space are represented by object positions.

(19) $$X_{GSA}^{i}=\left(d_{1}^{i},a_{2}^{i},a_{3}^{i},d_{4}^{i},d_{5}^{i},d_{6}^{i}\right),\;i=1,\ldots ,N$$

The mass value corresponding to the *i*th particle at iteration number t is called the non-normalized mass value and is represented by $m_{i}(t)$ [13].

(20) $$m_{GSA}^{i}(t)=\frac{f(X_{GSA}^{i})-f_{worst}(t)}{f_{best}(t)-f_{worst}(t)}$$

where $f_{worst}(t)$ is the overall worst fitness function value and $f_{best}(t)$ represents the overall best fitness function value. Therefore, $m_{GSA}^{i}(t)$ satisfies $m_{GSA}^{i}(t)\in [0,1]$, with the mass value corresponding to the best solution being equal to one and the mass value corresponding to the worst solution being equal to zero. The parameters $f_{worst}(t)$ and $f_{best}(t)$ are updated at every iteration as follows.

(21) $$f_{worst}(t)=max\{f\left(X_{GSA}^{i}\left(t\right)\right){\}}_{i=1,\ldots ,N}$$

(22) $$f_{best}(t)=min\{f(X_{GSA}^{i}(t)){\}}_{i=1,\ldots ,N}$$

where $x_{i}^{j}$ is the *j*th component of the particle position and N is the total number of particles. Each particle mass is updated and normalized at *t*th [13].

(23) $$M_{GSA}^{i}(t)=\frac{m_{GSA}^{i}(t)}{\sum_{k=1}^{N}m_{GSA}^{k}(t)}$$

The overall gravitational force ($F_{GSA}^{i}(t)$) acting on the *i*th particle is calculated using the gravitational law of force [13].

(24) $$F_{GSA}^{i}(t)=\sum_{j\in \{1,\ldots ,k_{b}\}}r_{j}G(t)\frac{M_{GSA}^{j}(t)M_{GSA}^{i}(t)(X_{GSA}^{j}(t)-X_{GSA}^{i}(t))}{||X_{GSA}^{i}(t)-X_{GSA}^{j}(t){||}^{r_{p}}+\varepsilon }$$

where $k_{b}$ is the number of selected best solutions, ||.|| represents the Euclidean norm, ε is a small value added to prevent singulairty, $r_{p}$ is the power value for the Euclidean distance between the two particles, G(t) is the gravitational constant, and $r_{j}\in [0,1]$ is a uniform and random value. The gravitational constant is updated at each iteration using the following equation [13].

(25) $$G(t)=G_{0}exp\left(-\beta \frac{t}{t_{max}}\right)$$

where $G_{0}$ has a constant real value and $t_{max}$ is the maximum value of the iterations of the algorithm. The acceleration term for each object is calculated according to Newton’s second law of motion by dividing the applied force to the *i*th mass by its mass value [13].

(26) $$A_{GSA}^{i}(t)=\frac{F_{GSA}^{i}(t)}{M_{GSA}^{i}(t)}=\sum_{j\in \{1,\ldots ,k_{b}\}}r_{j}G(t)\frac{M_{GSA}^{j}(t)\left(X_{GSA}^{j}(t)-X_{GSA}^{i}(t)\right)}{||X_{GSA}^{i}(t)-X_{GSA}^{j}(t){||}^{r_{p}}+\varepsilon }$$

where $A_{GSA}^{i}(t)\in R^{d}$ is the d—dimensional acceleration of the particles. The velocity value corresponding to each object is updated using the acceleration term and velocity vector [13].

(27) $$X_{GSA}^{i}(t+1)=X_{GSA}^{i}(t)+V_{GSA}^{i}(t+1)$$

## 3. Hardware Setup for the Experiment

**Figure 3:**
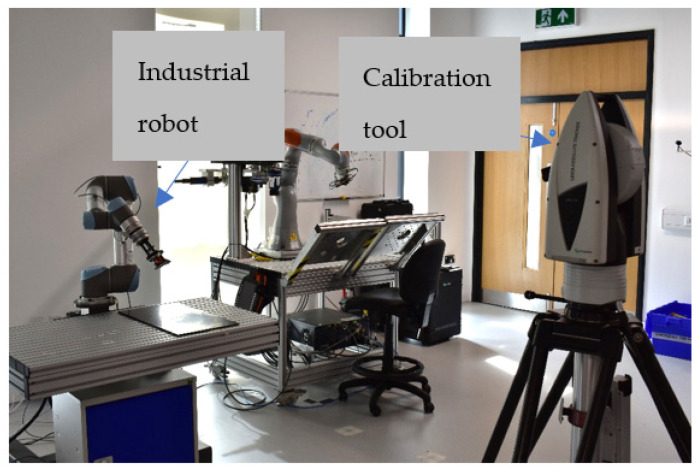
UR5 and the laser tracker.

The hardware setup for performing the experiment is explained under this section. To perform the calibration test, we need the industrial robot and independent calibration equipment, as shown in Figure 3. A detailed explanation of the hardware required to perform the calibration is explained in this section.

### 3.1. Industrial Robot: UR5

**Figure 4:**
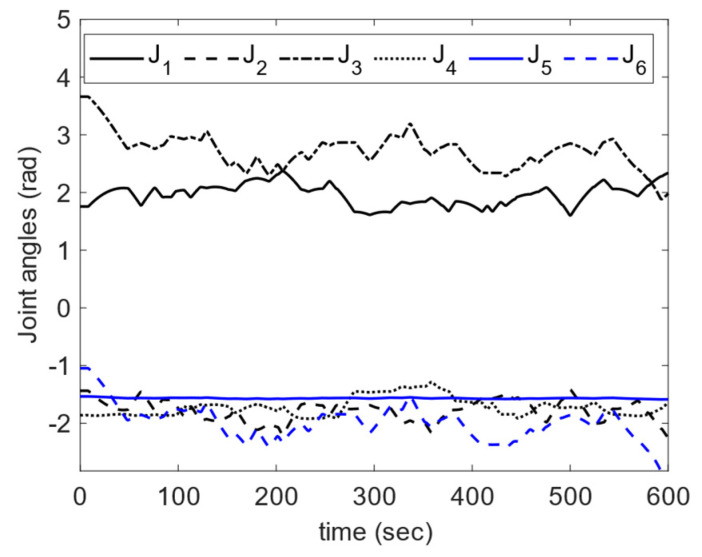
UR5 joint angle trends.

The Universal Robots—UR5 is a six-degrees-of-freedom (DOF) industrial robot capable of handling a 5 kg load with an angular velocity of 180°/s and an angular acceleration of $180{}^{\circ}/s^{2}$. The total reach of this robot without its grippers is 850 mm. Joint angle encoders and current measurements are performed in each joint. Joint angle measurements are used as the input to the robot FK to estimate its positions and orientations. However, the nominal position repeatability of UR5 is 0.1 mm. To collect joint angle values from UR5, its main controller is connected to a PC via LAN connectivity and a hub. The PC used for this experiment operates under Linux 18.04 OS, and the software interface for the robot is provided by ROS Melodic. The ROS driver that provides the connectivity is available through a GitHub webpage (https://github.com/UniversalRobots/Universal_Robots_ROS_Driver (accessed on 9 April 2023)). This ROS driver publishes a range of rostopics including joint angle values, joint angular velocities, and motor currents over time *@*125 Hz. In total, *38* waypoints are programmed for the UR5, which travels them linearly in 600 s. Figure 4 illustrates the robot joint angle values over time for all six joints of UR5. The position data gathered from the robot are resampled *@*10 Hz to match the laser tracker frequency.

### 3.2. Laser Tracker System

**Figure 5:**
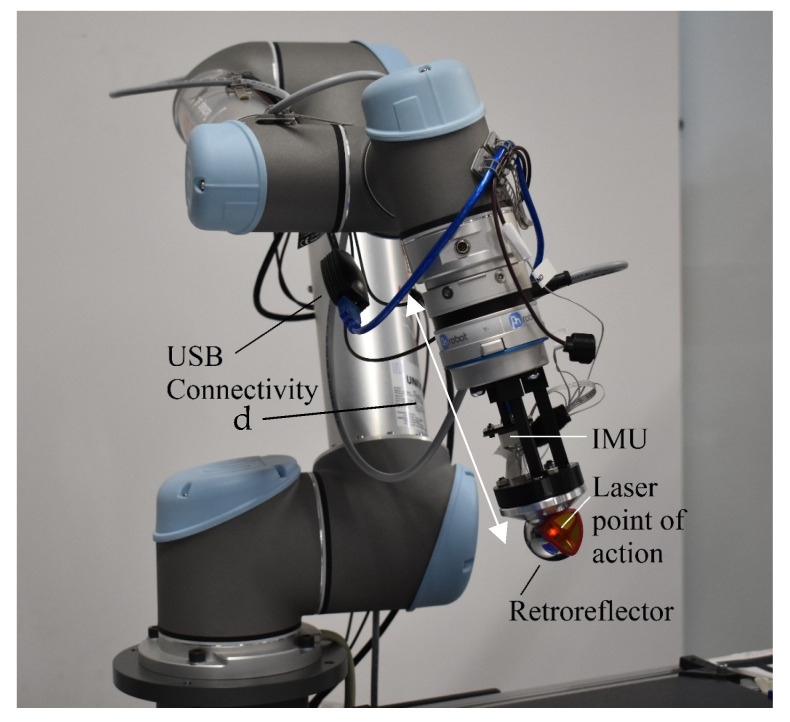
UR5 with a retroreflector mounted on it as the target for the laser tracker.

The laser tracker used in this experiment is AT960-MR, which is a portable dynamic 6DOF laser measurement system manufactured by Hexagon metrology GMBH, Wetzlar, Germany. This metrology equipment benefits from a single-class II laser source and is an IEC certified IP54 which guarantees ingress protection against dust and other contaminants for the unit. It benefits from Wifi connectivity, and in this work, it is set up to collect 10 data samples per second. Distance measurements are performed by using a retroreflector as the measurement target mounted on the UR5 end effector. This type of measurement system is a widely used one for precisely inspecting critical distances, locations, and surfaces [42] (see Figure 5). The target for the laser tracker is a precision Leica 1.5″ red ring reflector detectable through the laser tracker at a maximum distance of 60 m. In this work, the laser tracker is used within 2.8 m from the robot base. The reflector used in this experiment is using the principle of the corner cube. To reflect the beam, three plane mirrors at right angles to one another are used. The measurement point is the center of the reflector. To perform measurements, it is required to have a clear line of sight between the laser source and the retroreflector as the laser target.

Further specifications and environmental conditions of the laser tracker are presented in Table 2.

## 4. Experimental Results

### 4.1. Data Gathering

**Figure 6:**
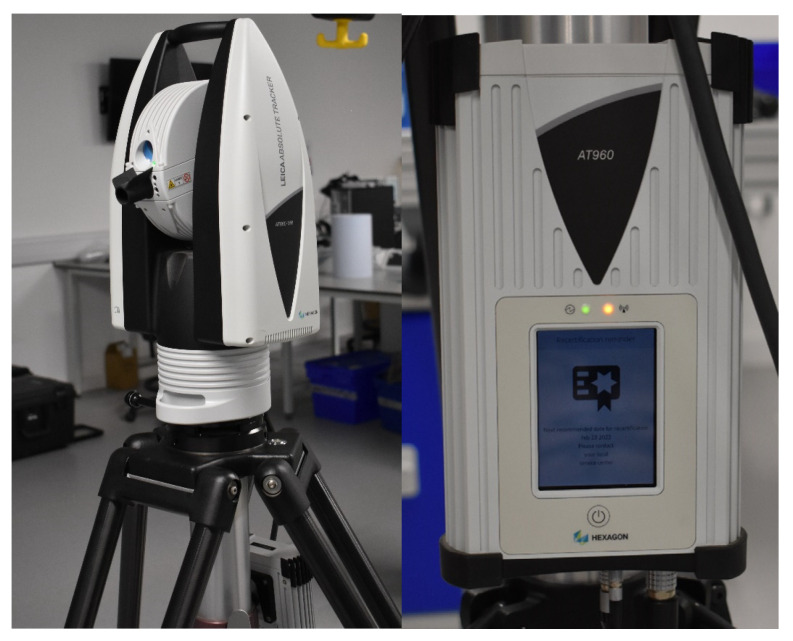
Laser tracker and its controller.

**Figure 7:**
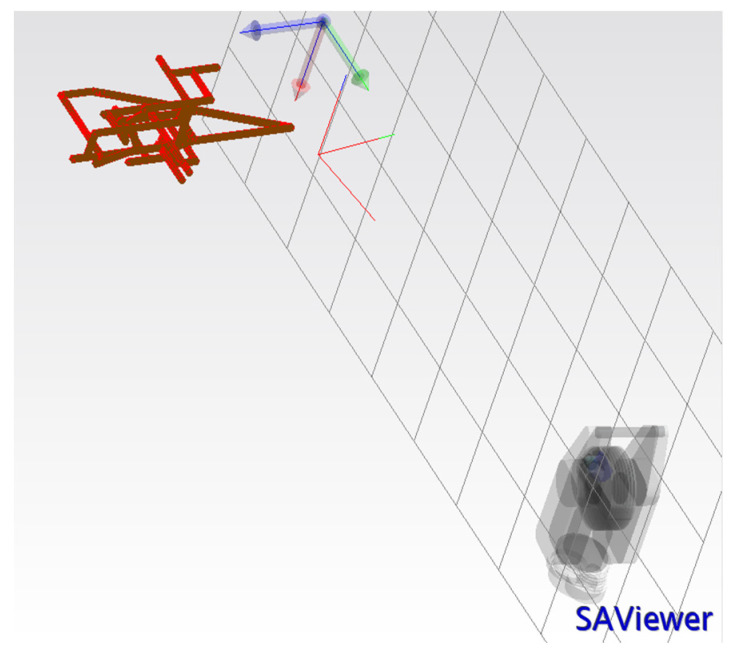
Screenshot of the points measured by the laser tracker system in Spatial Analyzer 2019 software.

**Figure 8:**
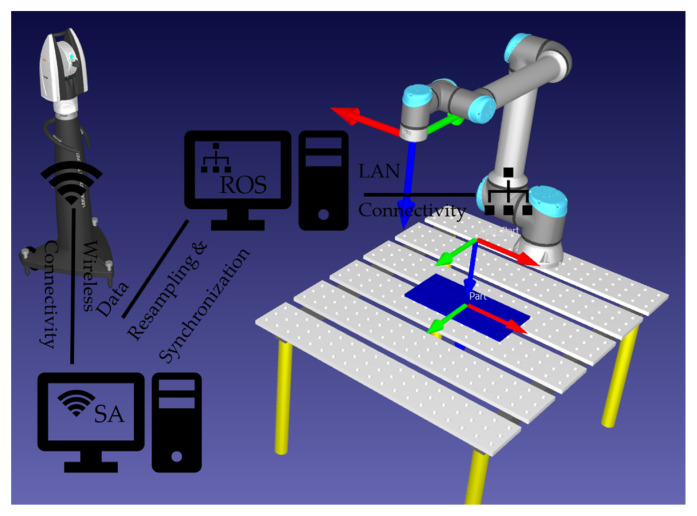
Connectivity required to perform the experiment.

To perform level II calibration on the industrial robot using the laser tracker system, the data flow graph as demonstrated in Figure 1 is followed. While data are collected from the laser tracker using Wi-Fi, the LAN network is used for collecting data from UR5. The ROS melodic package under Linux 18.04 is used to collect joint data including joint angle values, joint angular velocities, and joint efforts from UR5 @ ~125 Hz. The laser tracker data are collected using Spatial Analyzer 2019 software under a Windows 10 operating system (see Figure 6). It is required to shift, synchronize, and resample joint angle data for UR5 to match laser tracker data samples. Furthermore, to extract a near static data sample, the angular velocities less than 2 mm/s are considered. The total number of samples satisfying this angular velocity constraint is 209 points, from which 70% is used for training and the rest is used for testing purposes. The ROS melodic package under Linux 18.04 is used to collect joint data including joint angle values, joint angular velocities, and joint efforts from UR5 @ ~125 Hz. The laser tracker data are collected using Spatial Analyzer software under the Windows 10 operating system (see Figure 7). The connectivity required to gather the data from the laser tracker using SA and the data from UR5 using ROS melodic are illustrated in Figure 8.

### 4.2. Performance Measurement

There exist different performance indexes for validating the accuracy performance of models. In this paper, the mean absolute error (*MAE*) and standard deviation are used for performance measurement evaluation. These performance indexes are defined mathematically as follows.

(28) $${MAE}_{i}=\frac{1}{N}\sum_{k=1}^{N}\left|A_{ik}-F_{ik}\right|,i=x,y,z$$

(29) $$\sigma_{i}=\sqrt{\frac{1}{N}\sum_{k=1}^{N}{\left(A_{ik}-F_{ik}\right)}^{2}},i=x,y,z$$

where N is the number of samples, $\sigma_{i}$ presents the standard deviations in each dimension, $A_{ik}$ represents the data measured by the laser tracker in each of the three positional dimensions, and $F_{ik}$ represents the measurements by the FK of the robot in laser tracker coordinates in each of the three positional dimensions: x,y, and z.

### 4.3. Results

**Figure 9:**
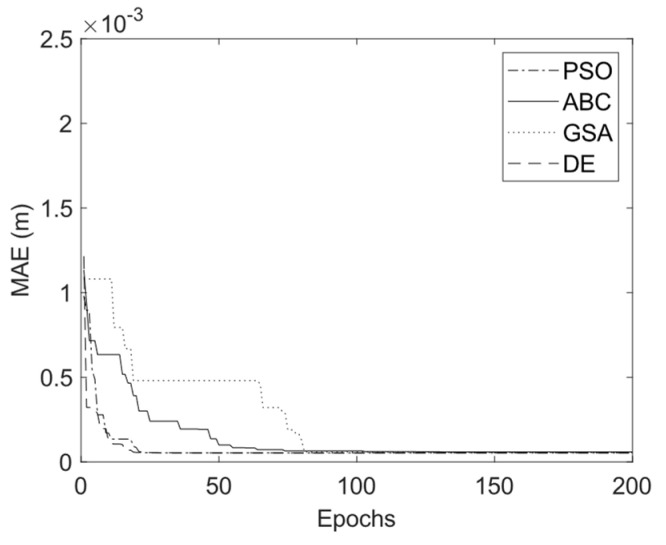
Convergence trend for PSO, ABC, GSA, and DE.

The parameters chosen for the three intelligent optimization algorithms of DE, PSO, GSA, and ABC are provided in Table 3. To have a better comparison, the population size for all three algorithms and the number of iterations are kept the same. Figure 9 demonstrates the convergence graph for all these optimization algorithms versus the iteration number. It is observed from the figure that DE converges faster than the other optimization algorithms of ABC, GSA, and PSO.

**Figure 10:**
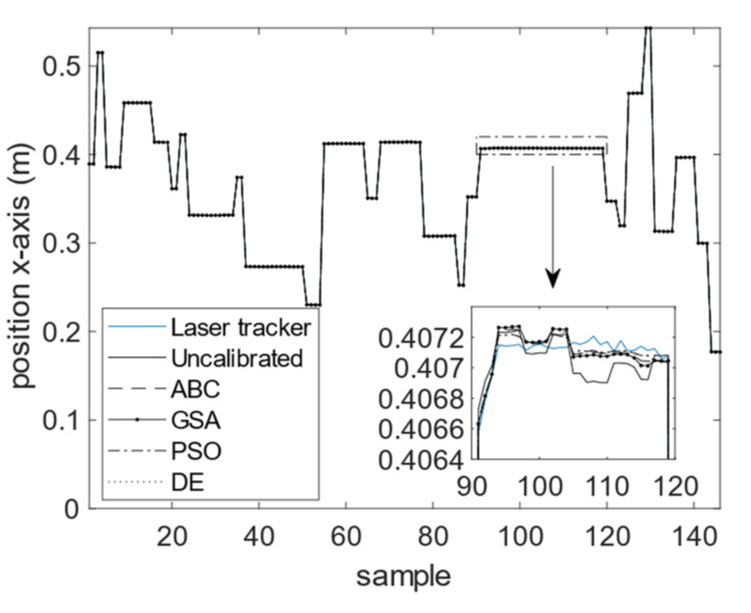
Calibration results in the *x*-dimension.

**Figure 11:**
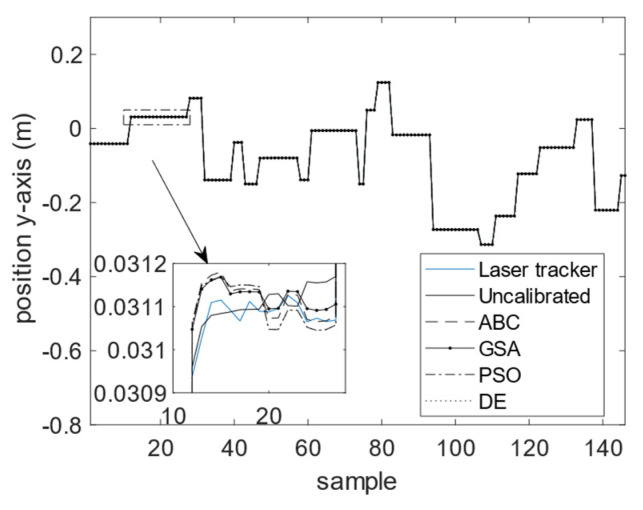
Calibration results in the *y*-dimension.

**Figure 12:**
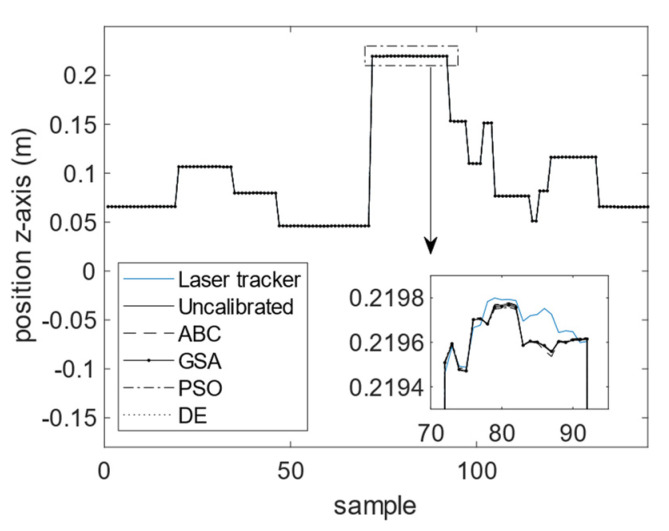
Calibration results in the *z*-dimension.

**Figure 13:**
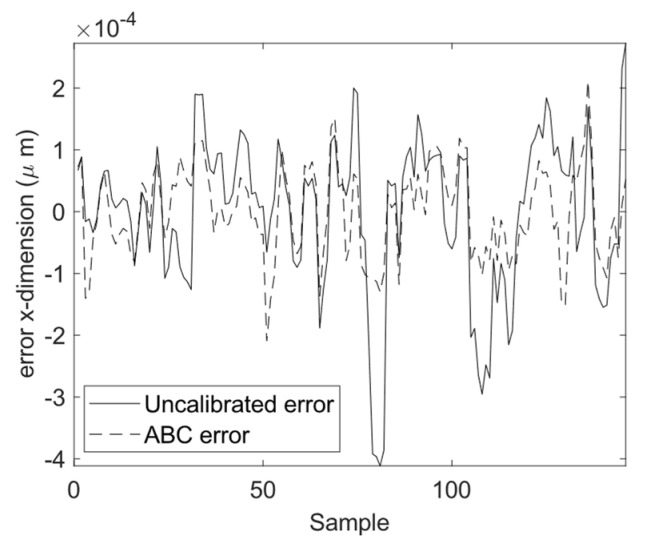
Calibration error in the *x*-dimension.

**Figure 14:**
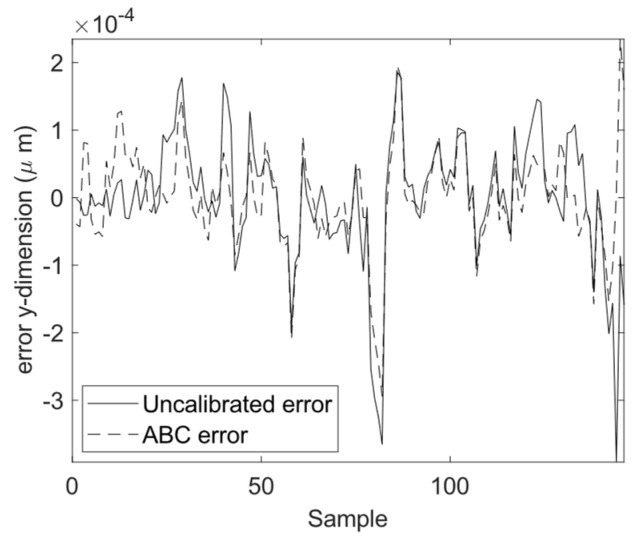
Calibration error in the *y*-dimension.

**Figure 15:**
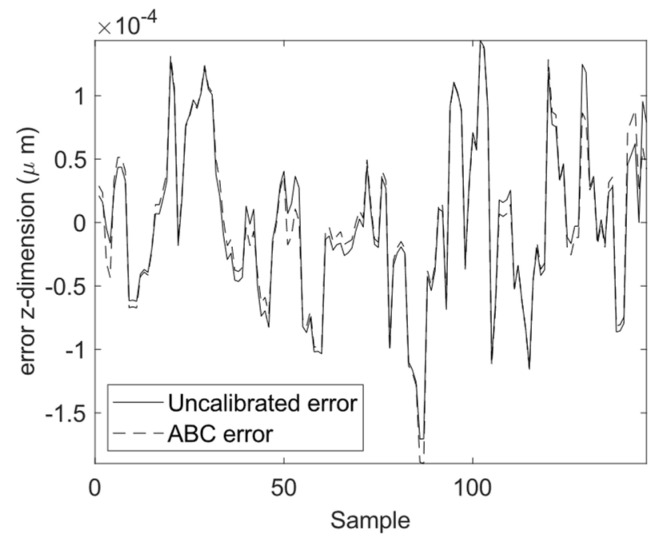
Calibration error in the *z*-dimension.

The calibration results for the four optimization algorithms of DE, GSA, PSO, and ABC are demonstrated in Figure 10, Figure 11 and Figure 12. As can be seen from these figures, the calibrated positions obtained through the four optimization algorithms are closer to the 3D precise measurements performed by the laser tracker in this experiment. The numerical values presented in Table 4 demonstrate the improvement made using the proposed calibration method. The MAE values associated with the calibrated FK using ABC in all three dimensions are reduced with respect to the uncalibrated version. The mean absolute value of error is reduced from 75.4 to 60.1 μm for the calibrated FK using ABC, which is a 20.3% improvement. It is further observed that the standard deviation of error is reduced from 100.6 to 76.1 μm. The improvement for the variance of error is 24.4%. The error trend associated with the industrial robot FK tuned by ABC as well as that of the original industrial robot FK are depicted in Figure 13, Figure 14 and Figure 15. As can be seen from the figures, error values for the calibrated FK are closer to zero as compared to the uncalibrated version in all three dimensions.

## 5. Conclusions

The sources of uncertainty in industrial robot FKs include manufacturing and assembly tolerances, dimension measurement errors, and environmental conditions. This paper deals with identifying more precise robot DH parameters to increase the positional accuracies of industrial robots. The proposed approach is a parametric calibration approach using a laser tracker. Therefore, not only is the proposed approach a precise approach for positioning robots, but the parameter values obtained using this approach also have physical meanings. To perform the calibration, a laser tracker system which is a non-contact metrology device is used. The laser tracker used in this experiment is a Leica AT960, with precision up to 3 μm/m, performing measurements 2.8 m away from the robot base. The industrial robot used in this experiment is a UR5, an industrial robot manufactured by Universal Robots. Using the four optimization algorithms of DE, PSO, ABC, and GSA, the tuning of industrial robot DH parameters is performed. It is observed that ABC outperforms DE, PSO, and GSA in terms of increasing the precision of robots. It is further observed that using the calibration approach proposed in this paper benefits from the ABC optimization algorithm results in decreasing the mean absolute value of error for the test data from 75.4 to 60.1 μm, which is a 20.3% improvement.

## 6. Future Works

In this paper, it is shown that the more accurate DH parameters can improve the accuracy of industrial robot FKs. The further accuracy improvement of industrial robots in future work requires online precision position feedback from measurement equipment and advanced computational inverse kinematic algorithms such as damped least squares. To increase the accuracy and convergence speed of the damped least squares algorithm, the DH parameter values obtained in this paper will be used. Furthermore, since the continuous monitoring of calibration and its validity over time is required, a mechanism for calibration monitoring is introduced to keep the position error within a valid interval. Such calibration monitoring system requires the continuous inspection and evaluation of the robot movements and positioning. This proposed approach will be implemented on more industrial robots to test the efficacy, performance, and implementability of the proposed methodology over a large number of industrial robots. The generalization of the proposed approach over a large number of industrial robots makes it possible to have statistical analysis of it and investigate the expected performance improvement using this approach in a more general form. Moreover, the calibration of industrial robots when the robot is operated at high speeds will also be considered. Contact approaches for industrial robot calibration [43,44] are usually lower-cost approaches for estimating more precise DH parameters of industrial robots. Motion sensors are another type of sensor which have already contributed to the precise calibration of industrial robots [45,46]. These types of sensors may perform orientation calibration with higher performance. The fusion of contact sensors for position measurements and inertia measurement sensors for orientation calibration would be considered in a future study.

## Figures and Tables

**Table 1:** The DH parameters of the 6DOF robot.

Link	q	d	a	α
1	$q_{1}$	$d_{1}$	*0*	π/2
2	$q_{2}$	*0*	$a_{2}$	*0*
3	$q_{3}$	*0*	$a_{3}$	*0*
4	$q_{4}$	$d_{4}$	*0*	π/2
5	$q_{5}$	$d_{5}$	*0*	−π/2
6	$q_{6}$	$d_{6}$	*0*	*0*

**Table 2:** Measuring equipment characteristics and specifications.

Environmental Working Conditions	IP54: The IEC-Certified Sealed Unit Guarantees Ingress Protection against Dust and Other Contaminants
Operating temperature	Wide operating temperature range of −15 to 45 degrees Celsius
Temperature compensation	MeteoStation: Integrated environmental unit monitors conditions including temperature, pressure, and humidity to compensate for changes.
ISO certification	ISO 17025
Connectivity	Wi-Fi and LAN
Detector features	Red ring reflector—1.5″ radius: 19.05 mm ± 0.0025 mm, centering of optics: <±0.003 mm, ball roundness: ≤0.003 mm, acceptance angle: ±30°, weight: 170 gr
Data output rate	Measurement rate of up to 1000 points per s
Distance accuracy	40 m in diameter and a 6DoF measuring volume of up to 20 m
Laser safety	Laser class 2
Distance to robot base origin	2.8 m

**Table 3:** Optimization parameters.

Algorithm	Parameter	Value
DE	F	0.55
	CR	2
	N	150
	max generation	300
GSA	$r_{p}$	1
	ε	$2.22\times 10^{-16}$
	β	20
	$k_{b}$	2
	$t_{max}$	300
	N	150
ABC	$S_{N}$	150
	$T_{max}$	300

**Table 4:** Mean absolute error values.

*Performance Indexes*		$\sigma_{i}$		*MAE*	
		Train	Test	Train	Test
*Uncalibrated* (μm)	X	125.5	117.8	95.9	90.3
	Y	94.3	105.1	64.0	77.5
	Z	64.2	73.5	50.7	58.4
	3D	97.9	100.6	70.2	75.4
*Calibrated* (μm) *Using ABC*	X	74.5	80.0	62.0	64.7
	Y	75.3	73.9	53.2	56.7
	Z	64.2	74.2	50.4	58.9
	3D	71.5	76.1	55.2	60.1
Calibrated (μm) *Using GSA*	X	79.9	86.0	65.8	71.1
	Y	70.9	66.8	51.3	52.6
	Z	64.1	74.2	50.7	59.0
	3D	71.9	76.1	56.0	60.9
Calibrated (μm) *Using PSO*	X	72.3	80.0	59.5	64.5
	Y	72.8	76.8	50.8	60.2
	Z	63.4	74.6	50.1	59.9
	3D	69.6	77.2	53.5	61.5
Calibrated (μm) *Using DE*	X	72.3	80.0	59.5	64.5
	Y	72.7	76.7	50.8	60.2
	Z	63.4	74.6	50.1	59.9
	3D	69.6	77.2	53.5	61.5

## Data Availability

Data would be available upon request on a personal contact with the corresponding author at the email address: mojtaba.ahmadiehkhanesar@nottingham.ac.uk.
